# Association of body shape index and body fat percentage with geriatric assessment outcomes

**DOI:** 10.1097/MD.0000000000043718

**Published:** 2025-08-22

**Authors:** Meryem Çakir, Olgu Aygün, Ayça Asma Sakalli, Yasemin Özkaya, Nafiye Ebru Terzi, Öykü Kahraman

**Affiliations:** a Department of Family Medicine, Izmir City Hospital, Izmir, Turkey; b Department of Family Medicine, Balikesir Atatürk City Hospital, Balikesir, Turkey.

**Keywords:** adipose tissue, aging, body composition, frailty, geriatric assessment

## Abstract

Body mass index loses predictive value with aging, while alternative indices like a body shape index (ABSI) may better reflect body composition changes. This study explores the relationship between ABSI, body fat percentage (%BF), and geriatric assessment outcomes. This cross-sectional study included 439 patients in home healthcare services. Geriatric assessment covered Barthel activities of daily living, mini nutritional assessment – short form, Mini-Mental State Examination (MMSE), geriatric depression scale-15, clinical frailty scale, strength, assistance with walking, rising from a chair, climbing stairs, and falls, and visual analog scale. Data were analyzed; regression models assessed ABSI and %BF effects. The mean age of the patients was 80.4 ± 8.1 years, the mean ABSI score was 0.125 ± 0.021, and the mean %BF was 33.5 ± 5.1. ABSI scores varied significantly across MMSE categories, with higher scores in the early-stage dementia group In regression models, ABSI was positively associated with MMSE and the Barthel index but negatively associated with visual analog scale. %BF was negatively associated with mini nutritional assessment – short form and frailty. ABSI was positively linked to cognitive function and daily activities, whereas %BF was negatively associated with nutritional status, frailty, and pain. Given the growing older population, integrating ABSI and %BF into clinical practice could enhance geriatric assessments and improve health outcomes.

## 1. Introduction

The World Health Organization predicts that by 2030, 1 in every 6 people globally will be considered older.^[1]^ With aging, height loss, muscle mass decline, and increased fat accumulation alter body composition, making body mass index (BMI) a less accurate measure of adiposity and health risk. Developed in 2012, ABSI (a body shape index), based on height, weight, and waist circumference, better reflects central abdominal fat.^[2]^ Studies have shown that ABSI demonstrates a stronger association with mortality risk compared to BMI or waist circumference.^[3]^ In geriatrics, increased fat with muscle mass is protective, while excess fat with low muscle mass harms physical performance, quality of life, and prognoses.^[4]^ It is known that body fat percentage (%BF) based on 4 caliper measurements is a good indicator.^[5]^ Studies suggest that ABSI may be associated with conditions important for geriatric assessment, such as cognitive function and osteoporosis, while %BF may be related to malnutrition.^[6,7]^ However, there is insufficient research explaining the relationship between ABSI and %BF and the outcomes of comprehensive geriatric assessments. The aim of this study was to investigate the relationship between ABSI, %BF and geriatric assessment outcomes.

## 2. Materials and methods

### 2.1. Study design

This retrospective cross-sectional study analyzed data from patients aged 65 to 100 receiving İzmir City Hospital Home Healthcare Services between April and December 2024. Ethical approval for the study was obtained from the Izmir City Hospital ethics committee.

### 2.2. Anthropometric measurements

The participants’ height (m), weight (kg), and waist circumference (m) were collected in a standardized manner by trained healthcare professionals. BMI and ABSI were calculated using the following formulas^[3]^:


$$\begin{array}{l} BMI:Weight(kg)•Height{\left(m\right)}^{-2} \end{array}$$

$$\begin{array}{l} {\text{A}BSI:WC(m)/[BMI}^{\text{2}/3}({\text{k}g/m}^{\text{2}})•{\text{H}eight}^{\text{1}/2}(\text{m})] \end{array}$$

In this study, BMI was categorized according to WHO criteria: underweight (<18.5 kg/m^2^), normal weight (18.5–24.9 kg/m²), overweight (25.0–29.9 kg/m²), and obese (≥30.0 kg/m²). In contrast, BF% and ABSI were evaluated as continuous variables and were not categorized in the analyses. There are age- and sex-specific reference ranges recommended in the literature for BF% (<35% for women and < 25% for men). ABSI values were interpreted based on typical ranges reported for older populations in the literature (0.110–0.130).^[2]^

### 2.3. Skinfold caliper measurements

The Harpenden Lange Skinfold Caliper (Cambridge Scientific Industries, Inc., Bethesda) was used by a trained professional to measure skinfold thickness at 4 sites – triceps, biceps, subscapular, and suprailiac – on the right side in a seated or upright position. 3 measurements per site were averaged. Body density was calculated using the Durnin and Womersley method, and BF% was determined via Siri equation.^[5]^

### 2.4. Tests used in geriatric assessment

Barthel activities of daily living (ADL) index: This scale assesses daily activities such as feeding, bathing, dressing, toileting, mobility, and transfers. Mini nutritional assessment short form (MNA-SF): This form evaluates nutritional status. Mini-Mental State Examination (MMSE): This test evaluates cognitive functions, with scores ranging from 0 to 30. Geriatric depression scale-15: this 15-item self-reported scale assesses depressive symptoms in geriatric patients, including energy, mood, hopelessness, loneliness, anxiety, and worthlessness. Clinical frailty scale: this scale evaluates frailty based on a clinician’s assessment, with scores ranging from 1 to 9. Strength, assistance with walking, rising from a chair, climbing stairs, and falls questionnaire: this scale is used for the rapid diagnosis of sarcopenia, characterized by the loss of muscle mass and function. Visual analog scale (VAS): the VAS is a subjective visual scale for assessing pain levels, ranging from 0 (no pain) to 10 (worst pain). Turkish validity and reliability studies of tests used in geriatric assessment are available.^[8–14]^

## 3. Statistical analysis

The Statistical Package for the Social Sciences (SPSS) 26.0 and AMOS 24 software programs were used for data analysis. Descriptive characteristics and disease status of the participants were presented as numbers and percentages. The normality of numerical data distribution was assessed using skewness and kurtosis values, and it was determined that ABSI and %BF variables followed a normal distribution. Independent sample *t*-test and one-way ANOVA Test were used to compare the descriptive characteristics and disease statuses of the patients with ABSI scores and %BFs. Differences between groups were examined using the Duncan multiple comparison test. Pearson correlation analysis was used to assess the relationship between ABSI scores, %BFs, and continuous variables, while Spearman correlation analysis was used for categorical variables. The effects of ABSI scores and %BFs on geriatric parameters were analyzed using regression analysis, which was performed with the AMOS 24 software. Significance levels of 0.05 and 0.01 were considered throughout the study.

### 3.1. Descriptive statistics

The mean age of the 439 patients included in the study was 80.44 ± 8.07 years, the mean ABSI score was 0.125 ± 0.021, and the mean %BF was 33.49 ± 5.05. In univariate analyses, women had significantly higher ABSI scores and %BFs compared to men (Table 1). Among the geriatric assessment tests, a significant difference was observed in ABSI scores based on MMSE categories, with particularly higher ABSI scores in the early-stage dementia group. %BF was found to be significantly lower in malnourished and frail individuals compared to other groups (*P* < .05; Table 2).

**Table 1:** Comparison of ABSI scores and body fat percentages according to patients’ descriptive characteristics and disease.

Descriptive characteristics		(%)	ABSI score Mean.±S.D.	*P*	BF% Mean ± S.D.	*P*
Gender	Male	39.9	0.119 ± 0.019	**.000**	29.52 ± 4.32	**.000**
	Female	60.1	0.130 ± 0.022		36.12 ± .57	
Age (years)	65–80	48.3	0.126 ± 0.022	.334	33.88 ± 5.31	.114
	81–100	51.7	0.124 ± .021		33.12 ± 4.78	
Dementia	No	90.0	0.125 ± 0.022	.801	33.46 ± 5.15	.755
	Yes	10.0	0.125 ± 0.022		33.71 ± 4.09	
Cerebrovascular disease	No	86.1	0.125 ± 0.021	.807	33.45 ± 5.08	.730
	Yes	13.9	0.126 ± 0.024		33.70 ± 4.90	
Coronary artery disease	No	75.2	0.125 ± 0.021	.506	33.4 ± 5.26	.803
	Yes	24.8	0.127 ± 0.024		33.59 ± 4.39	
Kidney failure	No	91.8	0.125 ± 0.021	.946	33.49 ± 5.08	.978
	Yes	8.2	0.125 ± 0.025		33.51 ± 4.77	
Cancer	No	90.2	0.125 ± 0.021	.722	33.60 ± 5.05	.156
	Yes	9.8	0.124 ± 0.027		32.45 ± 5.02	
Other diseases	No	36.0	0.126 ± 0.021	.575	33.66 ± 5.19	.598
	Yes	64.0	0.125 ± 0.022		33.39 ± 4.98	
BMI	Underweight	6.4	0.106 ± 0.011a	**.000**	27.96 ± 5.72a	**.000**
	Normal	39.4	0.110 ± 0.008a		31.55 ± 4.70b	
	Overweight	29.6	0.133 ± 0.016b		34.88 ± 4.44c	
	Obese	24.6	0.146 ± 0.022c		36.36 ± 3.50c	

Test statistic: Independent sample *t*-TEST (for two-group variables), one-way ANOVA (for variables with 3 or more), lettering: different letters indicate differences within groups (Duncan multiple range test).

Bold values indicate statistically significant results.

ABSI = a body shape index, BF% = body fat percentage, BMI = body mass index.

**Table 2:** Comparison of patients’ geriatric assessments with ABSI score and body fat percentage.

Variables		%	ABSI score Mean ± S.D.	*P*	BF% Mean ± S.D.	*P*
Barthel Index	Totally dependent (0–20)	14.4	0.14 ± 0.03a	**.050** *	32.74 ± 5.63	.357
	Severely dependent (21–61)	56.0	0.15 ± 0.02b		33.31 ± 5.05	
	Moderately dependent (62–90)	23.0	0.15 ± 0.03b		34.14 ± 4.82	
	Slightly dependent (91–99)	1.6	0.14 ± 0.01a		35.14 ± 2.39	
	Independent (100)	5.0	0.15 ± 0.03a		34.12 ± 4.78	
MNA-SF	Normal nutritional status (12–14)	29.4	0.14 ± 0.03a	**.036** *	34.45 ± 4.46a	**.000** **
	Risk of malnutrition (8–11)	44.9	0.15 ± 0.02ab		33.82 ± 4.59a	
	Malnutrition (≤7)	25.7	0.14 ± 0.03b		31.81 ± 6.00b	
MMSE	Severe dementia (<12)	41.7	0.14 ± 0.03	.230	33.48 ± 5.33	.446
	Moderate dementia (12–17)	15.0	0.15 ± 0.03		33.50 ± 5.09	
	Mild dementia (18–23)	27.1	0.14 ± 0.03		33.63 ± 4.85	
	Normal (≥24)	16.2	0.14 ± 0.03		34.16 ± 4.58	
GDS-15	No depression (0–4)	44.6	0.14 ± 0.03	.207	33.45 ± 5.02	.872
	Mild (5–8)	40.5	0.14 ± 0.02		33.88 ± 5.94	
	Moderate (9–11)	10.0	0.20 ± 0.00		34.56 ± 0.00	
	Severe (12–15)	4.8	0.14 ± 0.01		36.03 ± 2.61	
CFS	Not frail	10.3	0.14 ± 0.02	.119	34.43 ± 4.27a	**.026** *
	Pre-frail	39	0.15 ± 0.03		34.07 ± 4.74a	
	Frail	50.8	0.14 ± 0.03		32.86 ± 5.36b	
SARC-F	No risk (<4)	7.3	0.14 ± 0.02	.190	34.23 ± 4.86	.391
	At risk (≥4)	92.7	0.15 ± 0.03		33.43 ± 5.07	
VAS	Mild (<3)	73.6	0.14 ± 0.02	.415	33.77 ± 4.82	**.006** **
	Mild-moderate (3–6)	19.1	0.14 ± 0.2		33.41 ± 5.12	
	Moderate-severe (>6)	7.2	0.14 ± 0.01		30.81 ± 6.32	

Test statistic: independent sample *t*-test (for two-group variables), one-way ANOVA (for variables with ≥ three). Lettering: different letters indicate differences within groups (Duncan multiple range test).

Bold values indicate statistically significant results.

ABSI = a body shape index, BF% = body fat percentage, CFS = clinical frailty scale, GDS-15 = geriatric depression scale-15, MMSE = mini-mental status test, MNA-SF: mini nutritional assessment short form, ONS = oral nutritional solution, SARC-F = strength, assistance with walking, rising from a chair, climbing stairs, and falls questionnaire, VAS = visual analog scale.

* Statistical significance (*P* < .05).

** Statistical significance (*P* < .01).

### 3.2. Correlation analysis

In the correlation analysis, ABSI score was negatively associated with MNA-SF, VAS, Barthel index, and MMSE score (*P* < .05). The gender variable also showed a significant positive correlation with ABSI score (*P* < .05). Additionally, %BF showed a significant negative correlation with VAS, MNA-SF, and frailty, while it was positively associated with depression (*P* < .05; Table 3).

**Table 3:** Correlation analysis results between patients’ descriptive characteristics, disease status, and geriatric assessments with ABSI scores and body fat percentages.

Variables	ABSI score	BF%
ABSI	1.000	**.398** **
BF%	**.398** **	1.000
Gender	**.255** **	**.650** **
BMI	**.740** **	**.467** **
Barthel Index	**.117** *	0.089
MNA-SF	−.102*	−.175**
MMSE	**.114** *	0.047
GDS-15	0.053	**0.097** *
CFS	−0.004	−.126**
VAS	−.165**	−0.150**

Bold values indicate statistically significant results.

ABSI =: a body shape index, BF% = body fat percentage, BMI = body mass index, CFS = clinical frailty scale, GDS-15 = geriatric depression scale-15, MMSE = mini-mental status test, MNA-SF = mini nutritional assessment short form, VAS = visual analog scale.

* Statistical significance (*P* < .05).

** Statistical significance (*P* < .01).

### 3.3. Regression analysis

**Figure 1.**
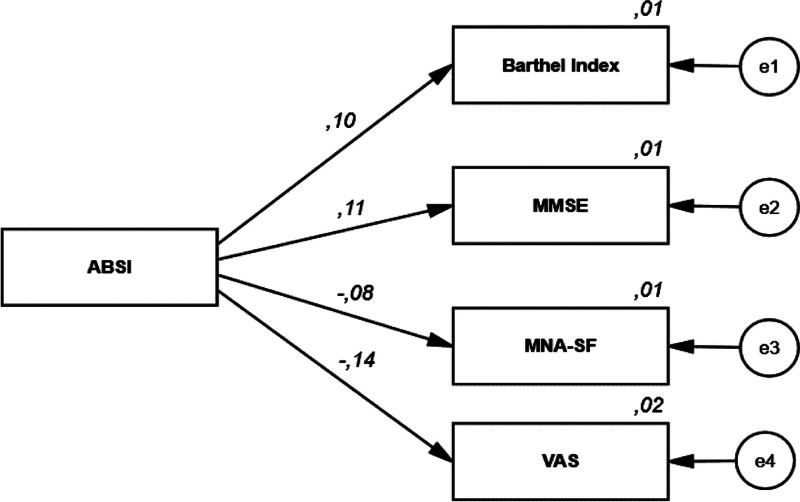
Regression analysis results examining the effects of patients’ ABSI scores on MMSE, Barthel index, MNA-SF, and VAS. ABSI = a body shape index, MMSE = Mini-Mental State Examination, MNA-SF = mini nutritional assessment – short form, VAS = visual analog scale.

**Figure 2.**
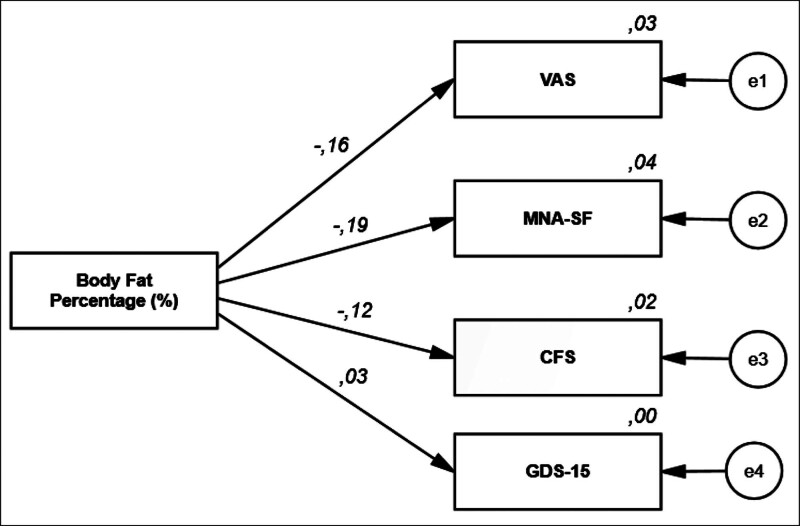
Regression analysis results examining the effects of patients’ body fat percentages on VAS, MNA-SF, CFS, and GDS-15. CFS = clinical frailty scale, GDS-15 = geriatric depression scale, MNA-SF = mini nutritional assessment – short form, VAS = visual analog scale.

Regression analysis showed that ABSI was positively associated with MMSE and Barthel index, and negatively associated with VAS (Table 4, Fig. 1). %BF was negatively associated with MNA-SF (β = −0.19, *P* = .000), frailty (β = −0.12, *P* = .009), and VAS (β = −0.16, *P* = .000) (Table 5, Fig. 2).

**Table 4:** Regression analysis results examining the effects of patients’ ABSI scores on MMSE, Barthel index, MNA-SF, and VAS.

Dependent variable	Path	Independent variables	*B*	*S.E.*	*β (Beta*)	*P*	*R²*
MMSE	<---	ABSI	47.50	19.90	0.11	**.017** *	0.013
Barthel index	<---	ABSI	121.94	55.72	0.10	**.029** *	0.011
MNA-SF	<---	ABSI	−2.78	1.63	−0.08	.089	0.007
VAS	<---	ABSI	−17.89	5.85	−0.15	**.002** **	0.021

Bold values indicate statistically significant results.

*B*: Unstandardized path coefficient, *β* (Beta): standardized path coefficient. Explained variance ratio in dependent factors:*R*^2^.

ABSI = a body shape index, BF% = body fat percentage, MMSE = mini-mental status test, MNA-SF = mini nutritional assessment short form, VAS = visual analog scale.

* Statistical significance (*P* < .05).

** Statistical significance (*P* < .01).

**Table 5:** Regression analysis results examining the effects of patients’ body fat percentages on MNA-SF, VAS, CFS, and GDS-15.

Dependent variable	Path	Independent variables	*B*	*S.E.*	*β (Beta*)	*P*	*R* ^*2*^
MNA-SF	<---	BF%	−0.03	0.01	−0.19	**.000** **	0.036
VAS	<---	BF%	−0.09	0.03	−0.16	.000**	0.027
CFS	<---	BF%	−0.02	0.01	−0.12	.009**	0.015
GDS-15	<---	BF%	0.01	0.01	0.03	.515	0.001

Bold values indicate statistically significant results.

*B*: Unstandardized path coefficient, *β* (Beta): Standardized path coefficient. Explained variance ratio in dependent factors: *R*^2^.

ABSI = a body shape index, BF% = body fat percentage, CFS = clinical frailty scale, GDS-15 = geriatric depression scale-15, MNA-SF = mini nutritional assessment short form, VAS = visual analog scale.

** Statistical significance (*P* < .01).

## 4. Discussion

This study aimed to investigate how ABSI and %BF are associated with multiple domains of geriatric assessment, including cognition, function, nutrition, frailty, pain, and psychological well-being. Among the key findings, the most notable was the positive association between ABSI and both cognitive performance and ADL, suggesting a potential role of ABSI in identifying older adults with preserved function. In contrast, %BF was negatively associated with frailty, malnutrition, and pain, emphasizing its utility in detecting risk in vulnerable subgroups. While each of these findings contributes to a broader understanding of anthropometric indicators in geriatric care, the relationships involving ABSI and cognitive/functional outcomes appear most novel and clinically meaningful within the context of this study.

In our study, the mean ABSI score of the patients was found to be 0.12 ± 0.02. It has been stated that the ABSI value for normal populations should be within the range of 0.07 to 0.09. Some studies show that ABSI scores increase with age.^[15]^ This finding is attributed to age related changes in body composition and ABSI’s role as a good indicator of it. A study of 944,769 patients aged 65 and older found that average ABSI scores were ≥ 0.1.^[16]^ Similarly, Zhang et al reported ABSI values exceeding 0.1 in some patients.^[6]^ Another study by Zhang et al confirmed that ABSI scores in older adults can exceed 0.1.^[17]^Similar to our study, older, multi-comorbid, or bedridden patients may have higher ABSI values than the general population. Future studies comparing ABSI in younger and older groups would clarify this.

This study on geriatric patients aged 65 + confirmed our hypothesis that ABSI and %BF are linked to geriatric assessment outcomes. ABSI showed a positive association with cognitive status and daily activities, while %BF was negatively related to nutrition, frailty, and pain. Interestingly, our findings suggest that higher ABSI scores are associated with better cognitive and functional performance, as reflected in increased MMSE and Barthel Index scores. While this may seem counterintuitive, several potential mechanisms may explain these associations. One such explanation is the obesity paradox, which describes a phenomenon in older adults where higher anthropometric measures (such as BMI or central adiposity indices) are paradoxically associated with improved survival or better functional outcomes. In geriatric populations, excess adipose tissue may provide metabolic reserves that protect against the adverse effects of aging, frailty, and subclinical disease processes. Moreover, selection bias may have contributed to this finding. Since our study was conducted among home-dwelling older adults receiving structured healthcare services, individuals with extremely low body mass or severe cognitive impairment may be underrepresented. Therefore, those with slightly higher ABSI might reflect a subgroup of older adults with relatively preserved physiological reserves.

In a study by Kashal et al on individuals aged 60+, no significant link was found between ABSI and cognitive impairment based on MMSE. However, they reported that higher BMI and waist circumference were associated with a lower risk of cognitive decline in older adults.^[18]^ Similarly, Li et al suggested that ABSI may be associated with poor cognitive function in individuals over 60 but emphasized that it is less effective than weight-adjusted waist index in reflecting cognitive impairment.^[19]^ Additionally, Sheng et al found no significant association between ABSI and cognitive impairment in their study of individuals over 50 years old without dementia.^[20]^ Some studies on BMI and waist circumference (WC) have suggested that higher values may provide protection against cognitive impairment.^[21,22]^ The literature on the relationship between ABSI and cognitive function is limited.

Abdominal obesity, particularly when accompanied by muscle mass loss, has been associated with functional decline and mobility limitations.^[23]^ A study on 2018 patients aged 65 + reported a negative correlation between WC and ADL.^[24]^ In contrast, in a study monitoring individuals aged 70 and over, found no significant relationship between ADL and BMI or WC.^[25]^ Our study found that in individuals over 65, higher ABSI scores were associated with increased Barthel scores. Research on the ABSI–ADL relationship is limited. Since ABSI measures central obesity, it may not always indicate functional decline, aligning with the “obesity paradox” often seen in older adults. This suggests that body composition and functionality in geriatrics are complex, and a single parameter may not fully capture clinical outcomes. The negative and significant relationship between %BF and MNA-SF observed in our study indicates that malnourished individuals had lower %BF. According to the European Society for Clinical Nutrition and Metabolism guidelines, malnutrition leads to a reduction in body fat mass.^[26]^ Similarly, López-Teros et al found that individuals with higher fat mass had a lower risk of malnutrition in a geriatric population.^[27]^ In older adults, assessing body composition can guide nutritional assessments and help determine patients’ dietary needs. Our study also found a significant negative relationship between %BF and VAS. Similarly, Sakai et al found that individuals over 65 years with nonspecific pain had higher fat percentages.^[28]^ However, a study on the Turkish population found no significant relationship between BMI and pain.^[29]^ Since body composition, metabolism, pain perception in older adults differ from younger populations, further research in geriatrics is needed to clarify this relationship. Frailty is associated with an imbalance between muscle and fat mass, with higher %BF often coinciding with lower muscle mass and increased fat accumulation.^[30]^ Yang et al linked both general and abdominal obesity with frailty.^[31]^ However, some studies have found no significant association between abdominal obesity and frailty, highlighting the need for further research on the relationship between %BF and frailty.^[32]^ Contrary to these findings, our study found that higher %BF was linked to lower frailty levels. An increase in %BF along with fat-free mass may reduce frailty risk. This hypothesis requires further research assessing %BF and fat-free body composition together. In our study, correlation analyses showed a positive relationship between %BF and depression, but this significance disappeared in multiple regression analyses. Studies on younger individuals suggest that a healthy fat distribution is associated with a lower risk of depression.^[33]^ Increased %BF may negatively affect self-perception, body image, and social interactions, potentially contributing to depression.^[34]^ An increase in %BF can impact both physical health and psychological well-being. However, further studies focusing on the geriatric population are needed to clarify this relationship. These relationships should also be interpreted in light of methodological considerations related to body fat measurement. %BF in our study was assessed using skinfold calipers, a method that may be less accurate in older individuals, particularly those with sarcopenia. In such cases, altered fat distribution and reduced compressibility of subcutaneous tissue may result in measurement bias. This could lead to either under or overestimation of %BF values and may have influenced the observed associations between body fat and clinical outcomes. Future studies using more precise body composition assessment methods (e.g., bioelectrical impedance analysis or dual-energy X-ray absorptiometry) could help validate these findings.

## 5. Limitations

Our study sample included home healthcare patients, limiting the generalizability of our findings to the broader older population. Additionally, skinfold caliper measurements may be less accurate in older individuals with low muscle mass or sarcopenia. In such cases, changes in fat distribution and reduced subcutaneous tissue compressibility may lead to measurement bias, potentially under or overestimating %BF. Furthermore, the low *R*² values reflect the multifactorial nature of health outcomes and underscore the importance of incorporating additional variables – such as inflammatory status – in future studies.

## 6. Conclusion

In this study, findings were obtained that help clarify the relationship between ABSI, %BF, and geriatric assessment outcomes. ABSI was positively associated with cognitive function and ADL, while %BF showed a negative relationship with nutritional status, frailty, and pain. With the global population aging rapidly, there is an increasing need for targeted diagnostic and therapeutic interventions for individuals over 65. ABSI and %BF are simple, practical tools for routine clinical use, offering valuable insights into geriatric assessments. Based on these findings, it is recommended that ABSI and %BF be incorporated into routine evaluations to support early identification of those at risk for cognitive impairment, functional decline, or malnutrition. Tailored strategies, including nutrition counseling, exercise programs, and body composition monitoring, may help improve outcomes and preserve independence in older adults.

## Author contributions

**Conceptualization:** Meryem Çakir.

**Data curation:** Olgu Aygün, Yasemin Özkaya, Nafiye Ebru Terzi, Öykü Kahraman.

**Formal analysis:** Yasemin Özkaya.

**Investigation:** Olgu Aygün, Öykü Kahraman.

**Methodology:** Meryem Çakir, Nafiye Ebru Terzi.

**Supervision:** Meryem Çakir, Olgu Aygün.

**Writing – original draft:** Meryem Çakir, Ayça Asma Sakalli.

**Writing – review & editing:** Ayça Asma Sakalli.
